# Generation of Multicellular Tumor Spheroids with Microwell-Based Agarose Scaffolds for Drug Testing

**DOI:** 10.1371/journal.pone.0130348

**Published:** 2015-06-19

**Authors:** Xue Gong, Chao Lin, Jian Cheng, Jiansheng Su, Hang Zhao, Tianlin Liu, Xuejun Wen, Peng Zhao

**Affiliations:** 1 Laboratory of Oral Biomedical Science and Translational Medicine, Department of Prosthodontics, School of Stomatology, Tongji University, Shanghai, P.R. China; 2 Institute for Biomedical Engineering & Nano Science, Tongji University School of Medicine, Tongji University, Shanghai, P.R. China; 3 Institute for Engineering and Medicine, Department of Chemical and Life Science Engineering, Virginia Commonwealth University, Richmond, Virginia, United States of America; Instituto Butantan, BRAZIL

## Abstract

Three dimensional multicellular aggregate, also referred to as cell spheroid or microtissue, is an indispensable tool for *in vitro* evaluating antitumor activity and drug efficacy. Compared with classical cellular monolayer, multicellular tumor spheroid (MCTS) offers a more rational platform to predict *in vivo* drug efficacy and toxicity. Nevertheless, traditional processing methods such as plastic dish culture with nonadhesive surfaces are regularly time-consuming, laborious and difficult to provide uniform-sized spheroids, thus causing poor reproducibility of experimental data and impeding high-throughput drug screening. In order to provide a robust and effective platform for *in vitro* drug evaluation, we present an agarose scaffold prepared with the template containing uniform-sized micro-wells in commercially available cell culture plates. The agarose scaffold allows for good adjustment of MCTS size and large-scale production of MCTS. Transparent agarose scaffold also allows for monitoring of spheroid formation under an optical microscopy. The formation of MCTS from MCF-7 cells was prepared using different-size-well templates and systematically investigated in terms of spheroid growth curve, circularity, and cell viability. The doxorubicin cytotoxicity against MCF-7 spheroid and MCF-7 monolayer cells was compared. The drug penetration behavior, cell cycle distribution, cell apoptosis, and gene expression were also evaluated in MCF-7 spheroid. The findings of this study indicate that, compared with cellular monolayer, MCTS provides a valuable platform for the assessment of therapeutic candidates in an *in vivo*-mimic microenvironment, and thus has great potential for use in drug discovery and tumor biology research.

## Introduction

Monolayer cell culture is traditionally used as *in vitro* model to investigate tumor behavior and identify effective antitumor therapies. Unfortunately, promising activities observed in two-dimensional (2-D) monolayer culture could not always be satisfactorily confirmed in animal studies or in clinical trials, because of the inability to replicate the extracellular microenvironment where cells reside in tumor tissues [1, 2]. Therefore, the development of powerful cell culture models that can help to bridge the gap between conventional monolayer cell studies and animal experiments is highly desirable. Three-dimensional (3-D) multicellular tumor spheroid (MCTS) models provide valuable tools for *in vitro* identification of potential anticancer drug targets [3–6]. Compared with conventional cellular monolayer, the heterogeneous architecture of MCTS more closely resembles the *in vivo* solid tumors. Large MCTS (>200 μm in diameter) is formed by concentric arrangements of peripheral proliferating cells, intermediate viable, but quiescent cells, and a central necrotic core [7–6]. In addition, the existence of extensive cell-cell and cell-extracellular matrix interactions, analogous to the *in vivo* tumor, promote the recovery of natural structures and functions of the original tissue biology [10–12]. The intercellular and extracellular link, with a concomitant elevation in the interstitial pressure, also provides a physical barrier to drug diffusion that contributes to drug resistance, which is not properly reflected in monolayer cell culture [13, 14]. Thus, MCTS may provide a valuable 3-D *in vitro* microtumor model for anticancer drug testing, which could be more predictive and more precise in mimicking an avascular tumor nodule.

Various techniques have been developed to generate MCTS. Traditionally, spheroids are formed using plastic culture dishes with nonadhesive surfaces [15, 16], or rotary cell culture systems [17, 18]. These culture systems allow single cells to spontaneously self-assemble, and eventually form multicellular aggregates. However, these techniques result in spheroids which usually display a broad size distribution. Uniformity of spheroid size is significant for obtaining highly reproducible results in drug assays and achieving a homogeneous and meaningful level of biological activities. The cell biology involving cellular functions within spheroids is strongly correlated with size [19]. Therefore, hanging drop cultures [20] and microfabricated microstructures [21, 22] are often used to overcome this problem. These methods compartmentalize the aggregation of individual spheroids to form uniform-sized spheroids, but have a limitation for mass production capabilities. The porous 3-D scaffold methods [23, 24], with physical support for cell self-assembly, are useful in controlling the spheroid size, however, difficulties in effective collecting and separation of spheroids from 3-D scaffolds remain. To facilitate the widespread implementation of MCTS in anticancer drug testing, new automated culture systems for the stable, scalable and reproducible production of MCTS with uniform characteristics are required.

In order to simultaneously study key cellular parameters that affect drug response and cell biology, we present a scalable and reproducible method for generating MCTS by an agarose scaffold with highly ordered micro-wells. It builds upon our previous microwell-based model [25], in which magnetic nanoparticles were used in directing the attachment and spatial organization of cells. These magnetic materials introduced the potential for cytotoxicity and their interferences with cell biology, and could not be separated from multicellular spheroids. In the present work, the prefabricated agarose scaffold allows for the rapid cellular assembly to form spheroids without any external forces, and enables quantitative and qualitative analysis of an individual spheroid or a single cell. This technique also offers a high yield of spheroids with excellent control over sizes. Spheroid culture of homogenous sizes and growth characteristics is significant for the consistent evaluation in drug assays. Furthermore, the described 3D agarose scaffold is completely transparent. This characteristic allows for simple monitoring of spheroid formation under traditional optical microscopy while avoiding specialized experiment equipment required for some other 3D culture systems. Specifically, the dimensions of master templates used for fabricating agarose scaffolds are tailored to fit in standard commercially available 6- and 24-well plates. These cell culture plates coated with micropore scaffolds on the bottom could be widely used in drug assays and other cell biology studies. Herein, human breast adenocarcinoma cell line, MCF-7, was used as a model culture system for micro-wells scaffold because of its practical usage for drug screening and well documented physiology. The formation of MCTS from MCF-7 cells was prepared using different-size-well templates and systematically investigated in terms of spheroid growth curve, circularity, and cell viability. MCF-7 spheroids with three different sizes were generated, and their resistance to doxorubicin was compared with monolayer cells. The relationships between spheroid size and drug resistance were evaluated. Drug penetration behavior, cell cycle distribution, cell apoptosis, and gene expression were also investigated in both monolayer culture and spheroid culture to determine how each affected drug resistance.

## Materials and Methods

### Materials

Doxorubicin hydrochloride (DOX HCl) was obtained from Sigma-Aldrich (St. Louis, MO, USA). Agarose was purchased from Invitrogen (Carlsbad, CA, USA). Fetal bovine serum (FBS), Dulbecco's phosphate buffered saline (PBS), penicillin/streptomycin, and trypsin/EDTA were obtained from Gibco (Carlsbad, CA, USA). Dulbecco's modified Eagle's medium (DMEM) was purchased from Hyclone (Logan, UT, USA). Live/Dead Cell Assay Kit, Quant-iT PicoGreen dsDNA Reagent and Kits, and the AlamarBlue Cell Viability Reagent were purchased from Invitrogen (Carlsbad, CA, USA). Complete protease inhibitor cocktail was purchased form Roche (Mannheim, Germany). Anti-GAPDH antibody and anti-CD44 antibody were purchased from Abcam (Cambridge, UK). Goat anti-rabbit IgG-HRP and the AnnexinV-FITC Apoptosis Detection Kit were purchased from Nanjing Jiancheng Bioengineering Institute (Nanjing, China). The Cell Cycle Detection Kit, the BCA Protein Assay Kit were purchased from Beyotime (Nanjing, China). The human breast adenocarcinoma cell line, MCF-7, was purchased from the Shanghai Institute of Cell Biology (Shanghai, China).

### Fabrication of agarose scaffolds with micro-wells

**Fig 1:**
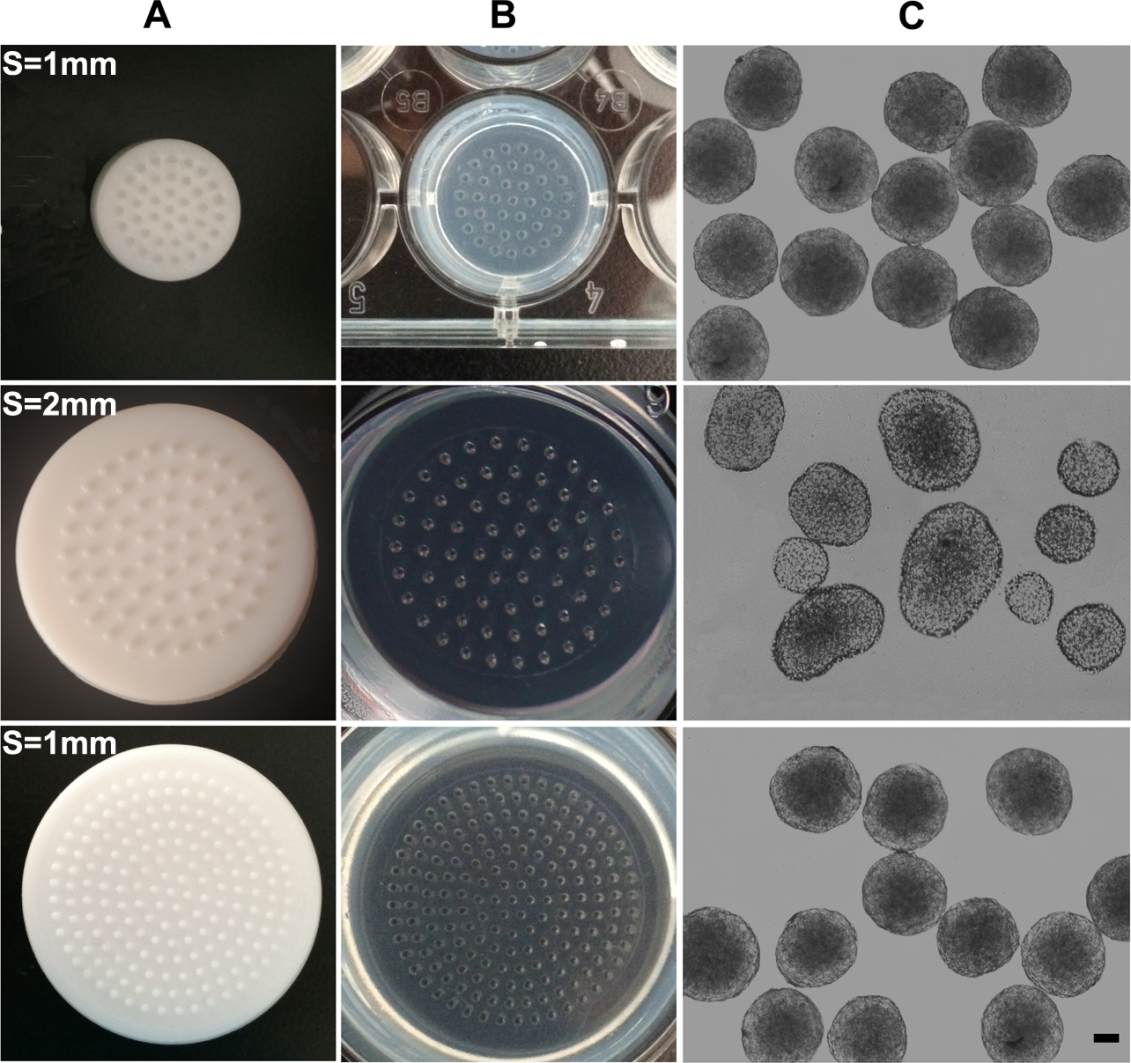
(A) Master templates used to fabricate micro-wells scaffolds. The master templates containing 175, 64 circular micro nipples suitable for 6-well plates. The master template containing 40 micro nipples suitable for 24-well plates. There are two kinds of template sizes and inter-pore spacings (S = 1mm, 2mm). (B) Produced micro-wells scaffolds. Agarose scaffolds containing uniform-sized micro-wells (~800 μm in diameter). (C) MCF-7 spheroids fabricated in different micro-wells scaffolds.

To produce multicellular tumor spheroids, master templates [25] were used to imprint micro-wells on agarose scaffolds, as displayed in Fig 1. Briefly, sterile 2% agarose in PBS was cast into cell culture wells, and then stamping of the agarose with the template containing micro nipples was done to fabricate uniform-sized micro-wells. After removing the master template, the cross-linked agarose scaffold with highly ordered micro-wells was coated on the bottom of a culture well. Culture plates should be exposed to ultraviolet light for 2 h to ensure sterility. Before the cell seeding, culture plates were rinsed with PBS and culture medium.

### Cell culture and MCTS formation

Human breast adenocarcinoma cells, MCF-7, were cultured in DMEM supplemented with 10% FBS, 100 IU/ml penicillin and 100 mg/ml streptomycin. The MCF-7 cells were incubated at 37°C in a humidified atmosphere of 5% CO2, and the culture medium was replaced every other day. At 90% confluency, MCF-7 monolayer cells were trypsinized into a single cell suspension, and then counted using a standard hemocytometer. The cell suspension was loaded into each well at densities ranging from 500 to 20000 cells per micro-well, depending on the desired multicellular spheroid size. The medium was exchanged to remove the nonadherent cells after 24 h of culture. The MCTS formation was monitored using optical microscopy (Nikon, Tokyo, Japan). To removal of MCTS from agarose micro-wells scaffolds for measurement, the spheroids were rinsed twice with PBS, aspirated gently by pipetting, and transferred to microcentrifuge tubes or cell culture plates.

### Tracking spheroid growth

Cells were seeded at three different concentrations (2000, 4000, and 8000 cells per micro-well), and cultured for 15 days as previously described. To examine the spheroid growth over time, ~30 spheroids for each cell seeding concentration were imaged every day using light microscopy (Nikon, Tokyo, Japan). The spheroid diameter was calculated by ImageJ (NIH, Bethesda, MD, USA). The spheroid area was measured by ImageJ, applying an image of known scale as calibration. As an indication of MCTS density, the solidity was also recorded. Furthermore, spheroid circularity was also measured using ImageJ.

### DNA quantification in MCTS

Cellular dsDNA from each cell remained constant. To trace the proliferation of MCTS, the cell number of the spheroid was determined by quantitative assessment of the amount of cellular DNA with Quant-iT PicoGreen dsDNA Reagent and Kits. The spheroids seeded at a density of 4000 cells per micro-well were collected for up to 12 days. For each sample, 1.0 ml of sodium citrate buffer solution, containing 100 mM NaCl and 50 mM sodium citrate, was added, and samples were then stored at -80°C until further analysis. The spheroids were lysed with sonication in sodium citrate solution. Then, 20 μl of the cell lysate was diluted with 80 μl Tris-EDTA buffer and incubated with 100 μl of the working solution of dsDNA reagent in 96-well plates for 2 to 5 min at room temperature. The DNA content of each sample was determined by fluorescence intensity of the mixed well with a fluorescence spectrophotometer (Thermo Fisher Scientific, Waltham, MA, USA), with excitation at 480 nm and emission at 520 nm. Data were analyzed by plotting fluorescence intensity versus DNA concentration.

### Cell viability within MCTS

Cell viability within the spheroids was monitored using the Live/Dead Cell Assay Kit. This assay was used to visually determine if cells within MCTS remained viable after spheroid formation. After removal of the spheroids from the agarose micro-wells scaffolds, 4-day-old, 10-day-old, and 12-day-old spheroids with different sizes, as previously described, were incubated with a solution containing 1 μM calcein AM and 4 μM ethidium homodimer-1 at 37°C for 30 min. After washing with PBS, spheroids were imaged by laser excitation of the sample at 488 nm and 561 nm. Spheroids were treated with 70% ethanol, and stained for use as live and dead cell controls.

### Drug resistance study in MCTS

Viable cell number was determined using the AlamarBlue Cell Viability Reagent. The assay was used to determine cell viability at each drug concentration relative to the wells without drug. DOX was selected for the study because of its clinical use in the treatment of metastatic breast cancer and other solid tumor malignancies. For 2-D culture, cells were seeded on flat-bottom 96-well plates at a density of 2×10^4^/cm^2^ and cultured for 1 day. For 3-D cultures, cells were seeded at three different densities (2000, 4000, 8000 cells per micro-well) on the agarose scaffolds, and were allowed to grow for 3 days under standard culture conditions. All samples were subsequently incubated in solutions of various concentrations of DOX. The cells were cultured for 3 more days and then a viability assay was performed following the manufacturer's protocol. A fluorescence spectrophotometer (Thermo Fisher Scientific, Waltham, MA, USA) was used to measure fluorescence intensity. Sigmoidal dose-response curves for 2-D and 3-D systems were then generated using GraphPad Prism 5.0 (San Diego, CA, USA), and the 50% inhibition concentration (IC_50_) value, which is the drug concentration at which half the cells were killed, was interpolated. The multicellular resistance index (MCRI), which represents the ratio of the IC_50_ cultured in a 3-D system to the IC_50_ cultured in a 2-D system, was also calculated.

### Visualization of drug penetration in MCTS

The distribution of doxorubicin within the MCTS was determined by a laser scanning confocal microscope (LSCM, Nikon, Japan) and by image analysis according to a previously published procedure with slight modifications [26]. 3-day-old spheroids with different sizes were harvested as previously described. For each group, approximately 30 spheroids were handpicked and placed in 1.5-ml Eppendorf tubes. Complete DMEM medium, with 30 μg/ml DOX, was added to the suspension of spheroids, and incubated at 37°C for 0.5 h, 2 h, and 4 h, respectively. The medium was then discarded and the spheroids were washed with PBS before observing by LSCM. The penetration area was determined by measuring the area where the drug signals were detectable, using Metamorph and ImageJ (NIH, Bethesda, MD, USA), and normalized by the area of each spheroid.

### Measurement of apoptosis in MCTS

The percentage of apoptotic and necrotic cells was determined by flow cytometry using the AnnexinV-FITC Apoptosis Detection Kit. For 3-D culture systems, 8-day-old and 12-day-old spheroids with different sizes were trypsinized to disperse into single cell suspension by gentle pipetting. For 2-D culture system, monolayer cells were routinely trypsinized. All the samples were then washed twice with ice-cold PBS. Finally, the resuspended cells were treated with AnnexinV-FITC Apoptosis Detection Kit following the manufacturer's protocol. Flow cytometry was performed with a flow cytometer (BD Biosciences, San Jose, CA, USA) equipped with FlowJo software.

### Cell cycle

Cell cycle distributions of the four different culture systems were determined by flow cytometry using the Cell Cycle Detection Kit according to the manufacturer's instructions. Briefly, the monolayer cells and 6-day-old spheroids with different sizes were trypsinized, washed with PBS, and immobilized in 1 ml 70% ethanol at 4°C for 12 h. Then, cells were washed with PBS, and incubated with 0.5 ml propidium iodide (PI) solution at 37°C for 30 min in the dark. Finally, samples were analyzed by flow cytometry (BD Biosciences).

### Western blot analysis

The expression of CD44 in monolayer cells and sphere cells was detected by standard western blot procedures. Primary antibodies used were anti-glyceraldehyde-3-phosphate dehydrogenase (GAPDH) and anti-CD44 monoclonal antibody, and secondary antibody was goat anti-rabbit IgG antibody conjugated with horseradish peroxidase (HRP). The monolayer cells and sphere cells (6-day-old, ~4000 cells per spheroid) were harvested and washed, and then lysed in RIPA buffer (Sigma-Aldrich) supplemented with complete protease inhibitor cocktail on ice for 30 min. Protein concentrations were determined by the BCA Protein Assay Kit. Equivalent quantities of protein from lysates were electrophoresed on 12% SDS-PAGE gels, and transferred to nitrocellulose membranes. The membranes were blocked in Tris-buffered saline (0.1% Tween-20 and 5% non-fat milk) for 1 h at room temperature, and incubated with primary antibodies overnight at 4°C. After extensive washing, the membranes were incubated with the HRP-labeled secondary antibodies. Signals were visualized by the Immune-Star Western C Chemiluminescence Kit (Bio-Rad, Hercules, CA, USA). The intensity of the bands was quantified with ImageJ software (NIH), and normalized to GAPDH expression levels.

### Quantitative RT-PCR measurement

Total cellular RNA of monolayer cells and sphere cells (6-day-old, ~4000 cells per spheroid), was extracted by Trizol (Invitrogen) according to the manufacturer's instructions. Equal amounts of RNA were reverse transcribed using a First-Strand cDNA Synthesis Kit (Toyobo, Osaka, Japan). Then, the qPCR reaction was performed on a Bio-Rad Real-Time PCR system (Bio-Rad) using SYBR Green RT-PCR Master Mix (Thermo Fisher Scientific). The relative quantity of gene expression compared with endogenous control GAPDH was calculated by the 2(-ΔΔCT) method [27]. The primer sequences were listed in Table 1.

**Table 1:** The primer sequences used for qRT-PCR analyses.

Gene	Forward primer	Reverse primer
**CD44**	5'- GATCATCTTGGCATCCCTCT -3'	5'- TGAGTCCACTTGGCTTTCTG -3'
**VEGF**	5'- GCAGCTTGAGTTAAACGAACG -3'	5'- GGTTCCCGAAACCCTGAG -3'
**GAPDH**	5'- CATCTTCTTTTGCGTCGCCA -3'	5'- TTAAAAGCAGCCCTGGTGACC -3'

### Statistical analysis

Data were expressed as mean ± standard deviation (SD). All experiments were done independently in triplicates. Statistical significance among the experimental groups was determined with Student's *t*-test. *P* values < 0.05 were considered statistically significant (*) and *P* values < 0.01 were considered extremely significant (**).

## Results

### Generation of uniform-sized spheroids on agarose scaffolds with micro-wells

**Fig 2:**
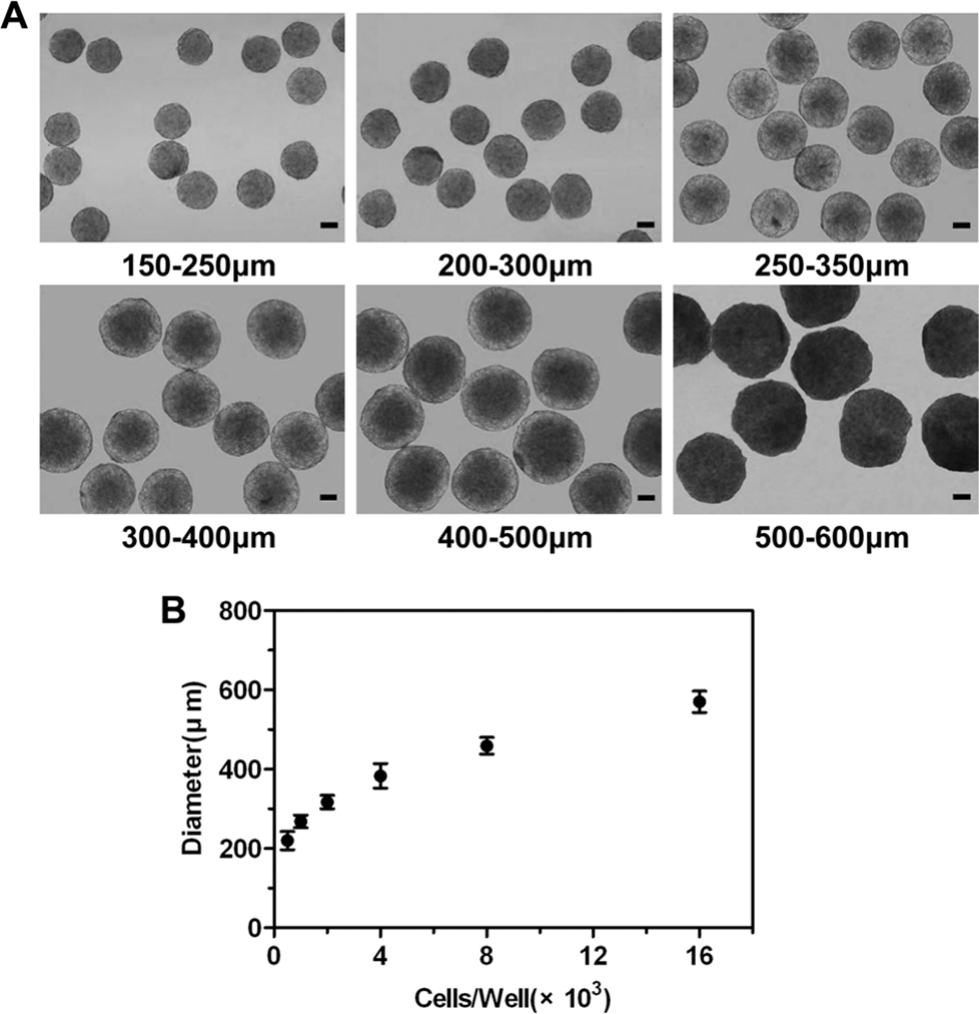
Generation of MCF-7 spheroids with different sizes by varying initial seeding cell concentrations. The diameters of spheroids were determined after 3 days of culture. (A) Spheroids with different diameters. Spheroid diameters of each group were highly uniform (SD ≤ 10%). Scale bars, 100 μm. (B) Spheroid size can be controlled by varying cell seeding number. Data represent the mean ± standard deviation (SD) from one experiment (n = 30).

Spheroids were generated using agarose scaffolds containing highly ordered micro-wells prepared by master templates. The agarose surfaces resisted cell adhesion and were well-suited for confining cell patterns during *in vitro* prolonged culture [15]. The micro-wells acted as centers of cell attachment for self-assembly and growth into spheroids. Different-size-well templates suitable for standard 6-well plate and 24-well plate, respectively, were used in this study, and inter-pore spacings (S = 1mm, 2mm) were varied for the demonstration of control over the resultant spheroid size (Fig 1). Regardless of the template size, spheroids of a homogenous size were reproducibly obtained using the micropore scaffolds with 1 mm inter-pore spacing, but spheroids generated on 2 mm inter-pore spacing scaffolds displayed a broad size distribution. The results indicated that the scalability of spheroid formation was not relevant with the template size, but the spacing among the pores. Therefore, micropore scaffolds with 1 mm inter-pore spacing were utilized for subsequent study. Fig 2A showed 3-day-old MCF-7 spheroids with different diameters. Spheroids diameters of each group were highly uniform (SD ≤ 10%). No breakups of the spheroids were observed after pipetting them from the micro-wells or transferring to cell culture plates, implying robust connections among the cells in the spheroids. To demonstrate reproducibility and scalability of spheroid formation, the dependency of cell seeding concentration on subsequent spheroid diameter was examined. Fig 2B showed the resulting trends for the MCF-7 cell line. The spheroid diameter increased with the increase of cell seeding concentration, indicating that the spheroids with the diameter ranging from 200−600 μm can be well controlled.

### Spheroid growth characterization

**Fig 3:**
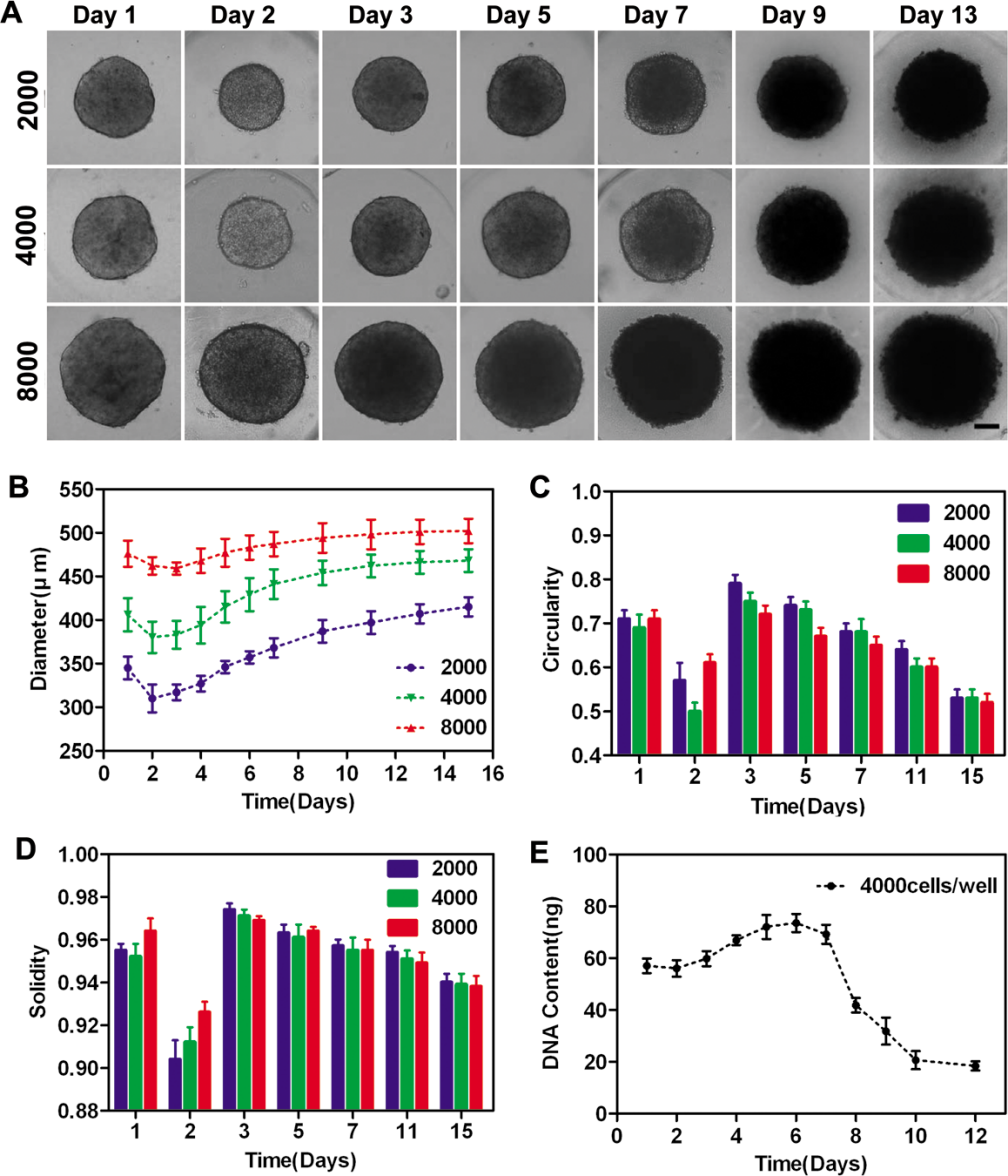
Spheroid growth characterization. (A) Spheroid growth assay. Representative images of spheroids of three different sizes. Scale bars, 100 μm. (B–D) The change of diameter, solidity, and circularity. Data represent the mean ± standard deviation (SD) of one experiment (n = 30). (E) Quantitative data for spheroid proliferation as determined by DNA content.

To monitor the growth of spheroids with different cell seeding concentrations, cultured in micro-wells, the diameter, solidity and circularity were investigated (see Fig 3). Fig 3A showed representative optical images of spheroids with different cell seeding concentrations (2000, 4000, and 8000 cells per micro-well). The morphological changes during spheroid formation involved several stages. In the initial stage, single cells spontaneously self-assembled to form cell aggregates in each micro-well and individual cells could be easily identifiable (day 1). Subsequently, cell aggregates began to fuse driven by intercellular interactions and contacts (day 2–3). Afterward, multicellular aggregates formed solid spheroids with smooth and continuous surfaces (day 3–5). In the final stage, the border of the spheroids became ruffled, suggesting the cell proliferation at the spheroid periphery (day 5–7), and individual cells were no longer distinguishable. After that, the spheroid size or cellular morphology no longer changed significantly. Fig 3B showed the change of mean spheroid diameter in the three groups. The spheroid diameter of all groups increased sequentially after an initial slight decrease. Thereafter, the diameter reached a plateau. Nevertheless, the plateau phase appeared earlier in groups of larger sizes, and the growth rate of smaller groups was higher than that of larger ones. The growth rate of spheroids with different sizes (2000, 4000, and 8000 cells per micro-well) was 31%, 24%, and 9%, respectively. The solidity and circularity histograms were shown in Fig 3C and 3D, and the changing trend of the two indicators was in agreement. The values of solidity and circularity increased after an initial remarkable decrease because of cellular reorganization and ECM secretion. Subsequently, a slight drop of spheroid solidity was observed over time, which possibly resulted from both cell apoptosis and the enlargement of the total spheroid area. The circularity also decreased over time because of cell proliferation at the spheroid periphery.

Quantitative data of cellular proliferation within spheroids were acquired by measuring the DNA content. As shown in Fig 3E, it took 6 days to reach the maximal DNA level, which declined rapidly after reaching a maximum, showing cell apoptosis and necrosis within the spheroids during the prolonged culture (see Table 2).

**Table 2:** Apoptosis of MCF-7 cells in spheroid culture and monolayer culture after 8 and 12 days of culture.

	2D	3-D(2000cells/well)		3-D(2000cells/well)		3-D(2000cells/well)	
		8 d	12 d	8 d	12 d	8 d	12 d
**Viable**	97.6±0.10	96.5±0.31*	92.9±1.00*	85.3±3.40*	53.6±3.95*	64.4±1.65*	29.2±3.46*
**Early apoptosis**	1.52±0.06	2.11±0.19*	6.50±0.75*	10.76±3.21*	43.4±3.97*	27.7±1.76*	54.7±2.24*
**Late apoptosis**	0.26±0.05	0.30±0.03	0.31±0.17	1.40±0.17*	2.65±0.24*	6.32±0.15*	15.23±1.51*
**Necrosis**	0.60±0.03	1.12±0.13*	0.29±0.07*	2.56±0.22*	0.43±0.12	1.53±0.37*	0.85±0.14

*Statistical significance (P<0.05) between 2-D monolayer cells and 3-D sphere cells.

### Cell viability within MCTS

**Fig 4:**
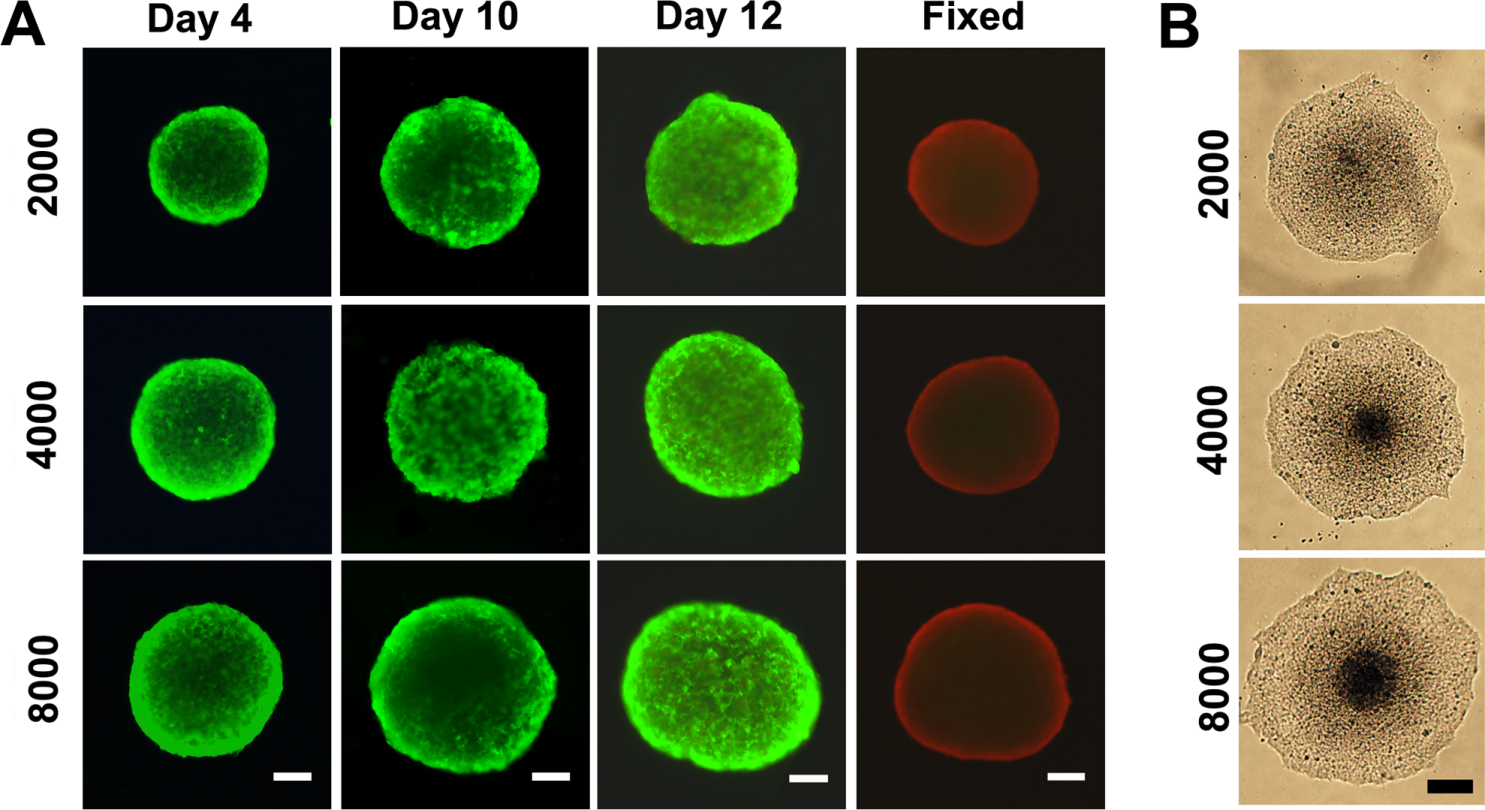
(A) Cell viability assay of spheroids with different sizes, using a live/dead staining with ethidium homodimer-1 and calcein AM. Viable cells appear as green, while nonviable cells appear as red. The fixed groups represent 10-day-old spheroids that have been incubated in 70% ethanol for 30 min, as a control. Scale bars, 100 μm. (B) After 24 h of culture, the 10-day-old spheroid of small-size group disassembled and fully spread, while spheroid of large-size group densely packed cells at the center of the spheroid, which appeared darker compared with the dispersed cells growing at the periphery. Scale bars, 200 μm.

The viability of cells within the spheroids was analyzed using Live/Dead Cell Assay Kit by fluorescent microscopy. The ethidium homodimer-1 staining was used to indicate a compromised cell membrane with subsequent binding to intracellular nucleic acids (red fluorescence), and calcein AM fluorescence demonstrated metabolically viable cells (green fluorescence). Fig 4A showed that cells within 4- and 10-day-old spheroids remained viable, however, cell death occurred. in all groups after 12 days of culture. The cell viability in the inner part of 10-day-old spheroids was also directly confirmed by transferring individual spheroids to 2-D cell culture plates, where the spheroids disassembled and fully spread after 12 h of incubation (see Fig 4B). The results indicated that cells at the center of spheroids remained viable and the capability of migration and repopulation were also retained.

### Penetration behavior of drug within MCTS

**Fig 5:**
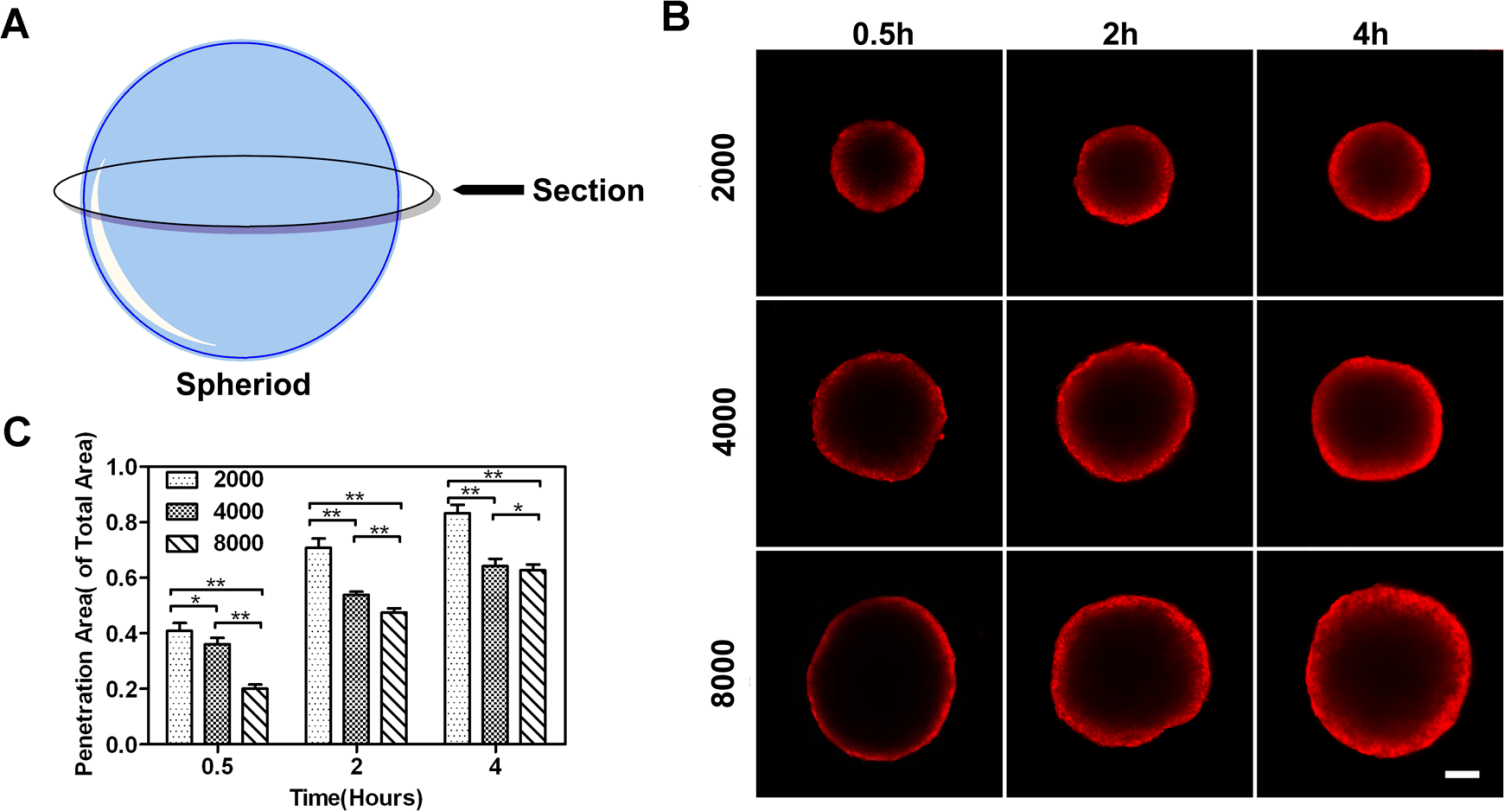
Penetration of DOX in MCF-7 spheroids. (A) Cross-sections at the middle of the spheroids were analyzed. (B) LSCM representative images of spheroid cross-sections, with different sizes, incubated with DOX for 0.5 h, 2 h, and 4 h. Scale bars, 100 μm. (C) Penetration area of DOX where the drug signals were detectable and normalized by area.

The restricted interstitial transport that blocks the diffusion of chemotherapeutic drugs into the target solid tumor tissue is a major *in vivo* barrier reducing drug efficacy [28]. 3-D spheroid culture model that mimics the extracellular microenvironment and diffusion processes that take place in actual solid tumors is used to achieve more accurate predictions of *in vivo* results in drug evaluation[29–31]. In the present study, we investigated the penetration behavior of DOX into spheroids. Fig 5B showed representative spheroid cross-sections observed by confocal microscopy. Fig 5C showed average penetration areas where the drug signals are detectable, normalized by the area of each spheroid. Apparently, a steady increase in penetration depth within MCTS was observed over time in all groups. For the large-size group (8000 cells per spheroid), the penetration was restricted to the outer few cell layers during the first 0.5 h. Moreover, the DOX fluorescence signal was found to penetrate approximately 100 μm from the spheroid boundary after 4 h treatment, and the spheroid core was still dark with no signals. Compared to the large-size group, the stronger DOX penetration for the small-size group (2000 cells per spheroid) was observed. A strong DOX signal spread from the periphery toward the core of MCTS over time. After 8 h of incubation, the signal had almost covered the entire MCTS. This fact suggested that the ability of DOX penetration in 3-D spheroids depends on their size.

### Drug cytotoxicity assays

**Fig 6:**
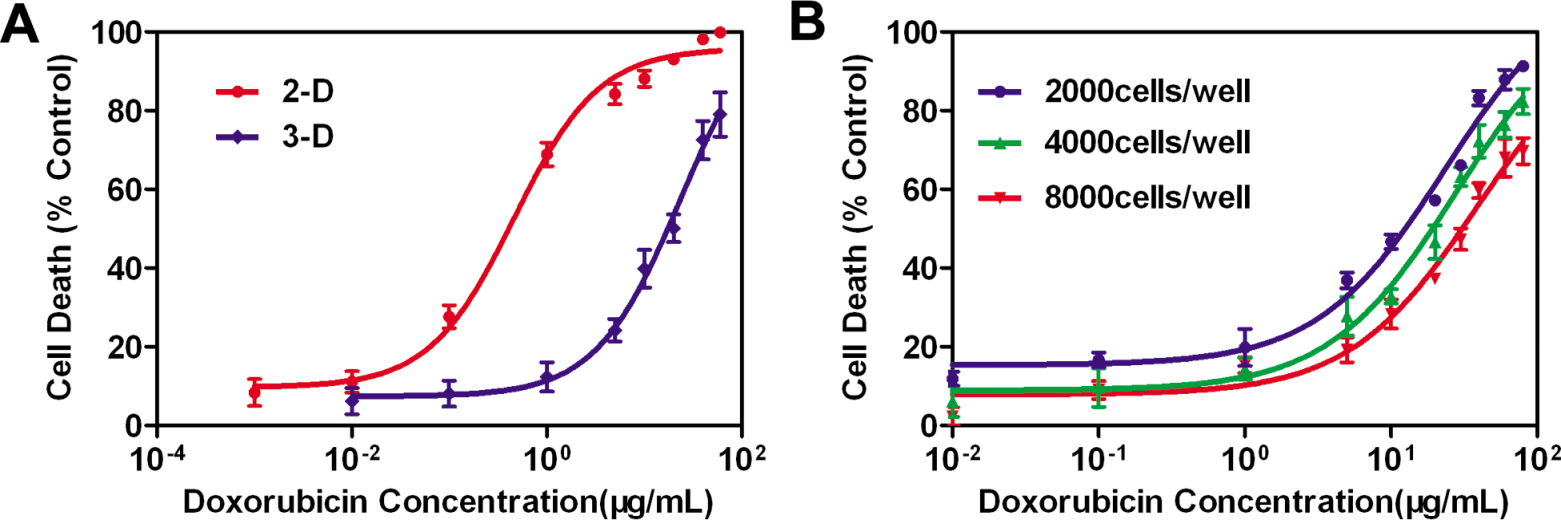
Assessment of drug cytotoxicity on agarose scaffolds with micro-wells. Data were expressed as mean ± standard deviation (SD) (n = 6). (A) Cytotoxicity testing of doxorubicin against MCF-7 cells in 3-D and 2-D cell culture models. (B) Size-dependent drug responses in 3-D cell culture models.

AlamarBlue Cell Viability assays were performed to determine drug cytotoxicity to monolayer culture and spheroid culture. Spheroids seeded with 2000, 4000, and 8000 cells per micro-well had diameters close to 300, 400, and 500 μm on day 3 (Fig 3B), respectively. Moreover, monolayer cells were simultaneously seeded in a 96-well plate as controls. As shown in Fig 6A, sphere cells displayed a higher drug resistance to DOX treatment in comparison to the 2-D controls. After 3 days of DOX treatment, the IC_50_ value obtained for spheroid culture was 23.2 μg/ml [95% confidence interval (CI) 13.8–39.2], relative to 0.46 μg/ml (95% CI 0.28–0.75) for the monolayer culture, which was consistent with previous reports [32, 33]. Toxicity assessments of DOX in 3-D spheroid culture were shown in Fig 6B, and IC_50_ and MCRI values were summarized in Table 3. The IC_50_ values determined with spheroids were approximately 50.3-fold, 60.0-fold, and 83.3-fold higher than that for monolayer cells. The data demonstrated that 3-D spheroid culture of MCF-7 cells were less susceptible to DOX than their 2-D counterpart. It was also notable that the spheroids of larger sizes exhibited higher drug resistance. This was consistent with the result we observed in the above penetration studies. The results indicated that this newly generated *in vitro* spheroid assay could be suitable and effective for drug screening.

**Table 3:** IC_50_ (μg/mL) and MCRI of systems assayed for drug resistance.

Culture system	Doxorubicin (95% CI)	MCRI	p-value
**2-D(20000/cm** ^**2**^ **)**	0.46 (0.28–0.75)	/	/
**3-D(2000cells/well)**	23.2 (13.8–39.2)	50.4	< 0.01
**3-D(4000cells/well)**	27.6 (17.1–44.7)	60.0	< 0.01
**3-D(8000cells/well)**	38.3 (19.2–76.2)	83.3	< 0.01

*P* values indicate significant differences between IC_50_ values in 2-D and 3-D cultures.

### Measurement of cell cycle and cell apoptosis

**Fig 7:**
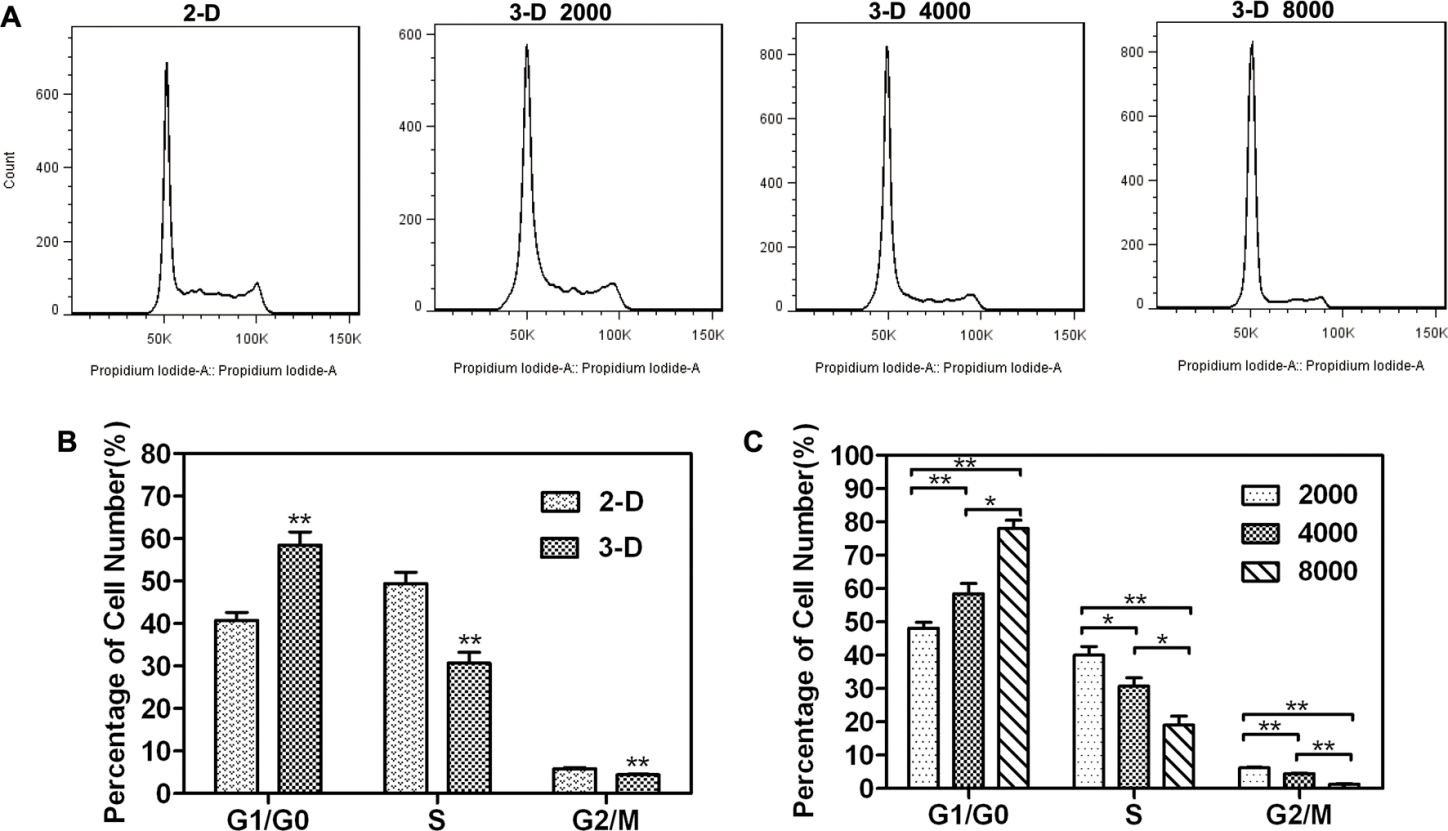
(A) Cell cycle analysis by flow cytometry. (B) Cell cycle distribution of MCF-7 cells in 3-D and 2-D models. (C) Cell cycle distribution of MCF-7 cells in spheroid cultures.

Cell cycle analysis by flow cytometry was shown in Fig 7. After 6 days of culture, the proportion of MCF-7 cells in G0-G1 phase, S phase and G2-M phase for 2-D monolayer culture and 3-D spheroid culture were 40.76%/58.48%, 49.44%/30.70%, and 5.81%/4.42%, respectively (see Fig 7B). For 3-D spheroid culture, the higher proportion of cells in G0-G1 phase suggested that more quiescent cells existed in spheroids, while the lower proportion of cells in S phase and G2-M phase suggested that cells grew and proliferated more slowly. This growth characteristics of MCTS might be a product of cell spatial organization and their heterogeneous architecture as previously reported [7–9]. Fig 7C showed the cell cycle distributions in spheroids with different sizes. The spheroids of smaller sizes displayed a higher proportion of cells in S phase and G2-M phase and a lower proportion of cells in the G0-G1 phase, indicating that spheroids of smaller sizes exhibited more proliferation capacity.

Apoptosis of MCF-7 cells in spheroid culture and monolayer culture were performed by an AnnexinV-FITC Apoptosis Assay, and the results were listed in Table 2. Trypsinized cells were treated with AnnexinV-FITC and PI which enabled to differentiate between live, early-apoptotic, late-apoptotic and necrotic cells [34]. The cell viability declined in 3-D culture in comparison to the 2-D control, and the proportion of cells in the early-apoptotic and late-apoptotic phase increased significantly, indicating the occurrence of hypoxia and nutrition deficiencies at the spheroid core. For 3-D culture systems, the cell viability within MCTS declined over time, and apoptosis was drastically increased during the prolonged culture. Moreover, spheroids of larger sizes displayed a higher proportion of apoptotic cells and a lower proportion of viable cells.

### Spheroid gene expression analysis

**Fig 8:**
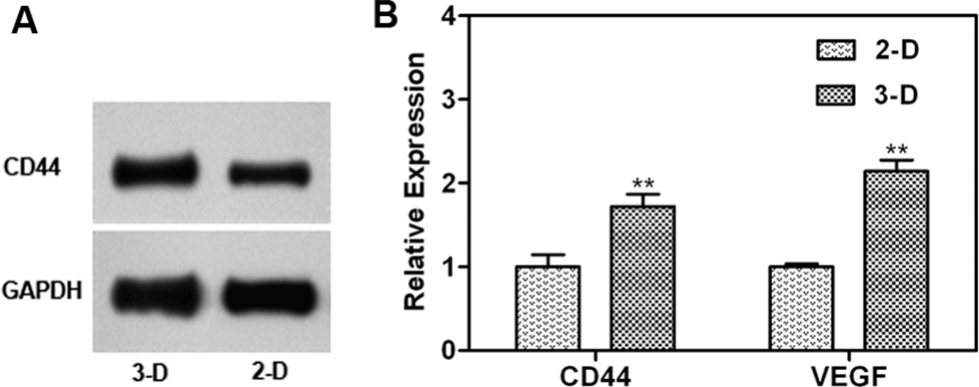
(A) The expression of CD44 in monolayer cells and sphere cells as measured by standard western blot procedures. (B) The expression of CD44 and VEGF in monolayer cells and sphere cells as determined by quantitative RT-PCR.

The complex spatial and physical structure of spheroids provides a physiological context that more closely mimics the tumor microenvironment. Multicellular drug resistance has been associated with cell contact inhibition and adverse microenvironmental conditions like hypoxia, acidic extracellular pH and nutritional depletion[35, 36]. Many reports have suggested the role of various cytokines in determining the drug response of cancer cells. For instance, as a result of both hypoxia and acidosis, angiogenic factors might be induced. Angiogenic factor, VEGF, a classic marker for hypoxic stress, has been shown to be correlated with chemoresistance[37, 38]. Moreover, CD44, a stem cell marker for breast cancer, has also been demonstrated to be responsible for the resistance to cytotoxic drugs[39, 40]. Thus, expression of the angiogenesis-associated gene (VEGF) and the adhesion molecule gene (CD44) in monolayer and spheroid culture were quantified and compared. In our study, the sphere cells expressed high levels of VEGF and CD44 in comparison to monolayer cells (Fig 8), which contributed to the elevated chemoresistance.

## Discussion

### Utility of the microwell-based spheroid culture model as a drug screening tool

Three-dimensional spheroid culture models are increasingly becoming essential tools in cancer research as they allow cellular responses that more closely mimic events occurring *in vivo*. Compared with monolayer cultures on conventional 2-D plastic surfaces, spheroid culture models provide a physiological context that more closely mimics the native tumor microenvironment[41–44], and thus are useful in the study of drug efficacy and toxicity. Nevertheless, methods currently available for MCTS generation are regularly time-consuming, laborious and difficult to generate spheroids with uniform sizes, thus causing poor reproducibility of experimental data and imeding high-throughput drug screening. To circumvent these problems, we reported a scalable and reproducible method to prepare uniform-sized spheroids using microwell-based agarose scaffolds, and demonstrated its applications in drug screening. This valuable 3-D *in vitro* microtumor model was more predictive and more precise in mimicking in vivo drug efficacy.

The model has many advantages. The prefabricated micropore scaffold allowed for the rapid cellular assembly to form spheroids without any external forces, and enabled quantitative and qualitative analysis of an individual spheroid or a single cell. This model also offered a high yield of spheroids with excellent control over sizes. Standardization of spheroid size is critical for the reproducible evaluation in drug assays as cell biology and diffusion limitations vary with the size of MCTS. Furthermore, the transparent agarose scaffolds allowed for monitoring of spheroid formation by traditional optical microscopy, and the generated spheroids with compact and rigid structure could be recovered from scaffolds easily without affecting the physical characteristics. Wan Yong Ho et al. reported a spheroid culture model for drug screening using the MTT Assay, in which a non-adherent agarose surface was pre-coated in 96-well plate for producing spheroids with uniform sizes [45]. However, external force was applied in directing the attachment and spatial organization of MCF-7 cells, which may introduce the interference with cell biology. Moreover, the cell suspension should be seeded into each well for generating an individual spheroid. It was cumbersome and time-consuming for massive cell spheroid formation. In our study, single cells spontaneously self-assembled to form spheroids on agarose scaffolds prepared by master templates without any external forces, and the agarose scaffolds with ordered micro-wells also allowed for large-scale production of spheroids. The dimensions of master templates were tailored to fit in 6, 24 well plates, and these modified cell culture plates pre-coated with agarose scaffolds could be widely used in drug assays.

In our study, agarose has been used because of its high water content and biocompatibility. Because this agarose surface strongly resists cell adhesion, the cells settle from suspension into discrete populations of equivalent numbers for cultures of uniform-sized spheroids. Using MCF-7 cell lines derived from breast carcinoma, we demonstrated the ability to accurately control the size of multicellular tumor spheroids based on cell seeding number. MCF-7 spheroids were prepared ranging from 200 to 600 μm in diameter based on initial cell seeding number (Fig 2B). Additionally, the templates tailored to fit in standard 6- and 24- well plates demonstrated the mass production capabilities for spheroids formation (Fig 1). To demonstrate the wider applicability of this micro-wells model, MCTS could also be formed from other cancer cell types (e.g. ovarian cancer, liver cancer, and prostate cancer cells), and other stromal cells (e.g. endothelial cells for vascularized tumor models) for anticancer drug testing (data not shown).

Herein, human breast adenocarcinoma cell line, MCF-7, was used as a model culture system for the agarose scaffold because of its practical usage for drug screening and well documented physiology. The drug cytotoxicity assays demonstrated that cells grown as MCTS developed multicellular chemoresistance. In the dose-response experiment involving spheroid culture, the IC_50_ value was 23.8 μg/ml, approximately 50-fold higher than that of monolayer culture (Fig 6A and Table 3). The 3-D spheroid culture model exhibited a greater resistance to anticancer drug than 2-D cultures, which was consistent with the results observed in previous studies [46–48]. Therefore, this 3-D MCTS model could better predict the clinical activity of candidate chemotherapeutics and may also be useful in cancer research.

### Understanding the basis of increased drug resistance

In this study, the phenomenon of multicellular spheroids displaying elevated chemoresistance to anticancer drugs was attributed to a decreased drug penetration, cell cycle arrest, and altered gene expression conferring drug resistance.

Compared with monolayer culture, 3-D spheroid culture models have an additional dimension of extensive cell-cell interactions, analogous to the *in vivo* solid tumor. The intercellular interactions with high interstitial fluid pressure provide a physical barrier to limit drug diffusion, resulting in an enhanced resistance to chemotherapeutics [13, 14]. Our study showed that DOX signal was restricted to the outer layer of spheroid in the large-size group (8000 cells per spheroid) after 4 h incubation. The core of the spheroid appeared dark with no drug signals (Fig 5B). The elevated chemoresistance might be associated with a restricted uptake and penetration of anticancer drug into the deeper layers of MCTS. This was consistent with spheroid permeability data obtained in previous studies [49]. Spheroids with 3-D cell contacts are more predictive of *in vivo* drug responses than monolayer models.

MCTS (>200 μm in diameter) exhibit a heterogeneous architecture with an inner necrotic core, intermediate quiescent region, and a peripheral proliferating region [7–9]. The gradation of oxygen within MCTS provides an inner hypoxic core, similar to the hypoxic regions found within solid tumors. Nevertheless, a lot of antitumor agents exerted limited toxicity against slowly proliferating or quiescent cells and were less cytotoxic in hypoxic and acidic microenvironment of tissues [50,51]. As revealed by the cell cycle distribution data, the proportion of MCF-7 cells in the G0-G1 phase, S phase, and G2-M phase for 2-D culture and 3-D culture was 40.76%/58.48%, 49.44%/30.70%, and 5.81%/4.42%, respectively (Fig 7B), showing that the cell proliferation was much slower in spheroids. This explained the elevated chemoresistance, and thus promotion of cell survival during drug treatment of the slowly proliferating spheroids.

The complex spatial and physical structure of spheroids affects the signal transduction pathways, and ultimately changes the cellular phenotypic expression and cell behavior. CD 44 is a cell membrane proteoglycan that mediates cell-cell and cell-matrix adhesion [52, 53]. CD 44 is also the most widely used marker to identify breast cancer stem cells [54], and has been shown to be responsible for the resistance to cytotoxic drugs[39, 40]. In our study, the gene expression of CD 44 was upregulated after spheroid formation (Fig 8), demonstrating that MCTS presented a spatial structure with extensive cell-cell interactions, which contributed to the elevated chemoresistance. Moreover, VEGF has also been shown to be correlated with chemoresistance. Tumor cells respond to hypoxia and acidosis by stimulating neovascularization and inducing the expression of angiogenic factors such as VEGF [55]. Many earlier studies reported that inhibiting the activity of VEGF could lead to elevated chemosensitivity, increased expression of proteins that involved in the induction of apoptosis [37, 38], and thus reduced cell survival. The results showed that there was a significantly higher expression of VEGF in spheroids (Fig 8), which resulted in an enhanced resistance to chemotherapeutics.

### Size-dependent drug resistance of spheroids

Herein, we examined a scalable method to prepare uniformly sized spheroids, and demonstrated applications in drug screening. Using homogeneous spheroids with three different sizes, we investigated the dependence of drug cytotoxicity with spheroid size (i.e. 300, 400, and 500 μm), and compared the results with traditional 2-D cultures. To our knowledge, this is the first study to show the relationships between spheroid sizes and drug resistance. As shown in Fig 6B and Table 3, the spheroids of larger sizes exhibited higher drug resistance. The IC_50_ values of three culture systems were approximately 50-fold, 60-fold, and 80-fold, respectively, higher than that of monolayer cells.

The drug permeability, cell cycle distribution, and hypoxia state are all factors that associated with distinctive chemoresistance. The penetration capability of anticancer drugs can be influenced by MCTS size. In Fig 5B, the stronger doxorubicin penetration was observed within the smaller MCTS. Additionally, the proportion of cells with a characteristically low proliferative rate increased within the larger MCTS (Fig 7C), as a larger spheroid should be more hypoxic. Because antitumor drugs cannot exert effective toxicity against these slowly proliferating or quiescent cells, larger spheroids showed a higher chemoresistance. These data suggested that 3-D spheroid drug sensitivity depended on size. The potential to precisely control the spheroid size by a microwell-based culture model provides an interesting opportunity for tumor biology research.

## Conclusions

In summary, a new microwell-based spheroid culture model can provide massive uniform spheroids with a narrow size distribution. Cells grown as MCTS developed multicellular chemoresistance similar to the tumor with decreased drug penetration, cell cycle arrest, hypoxia, and altered gene expressions. Drug resistance was demonstrated to be strongly dependent on the size of the MCTS. This study will contribute to the development of 3-D MCTS models, which can provide valuable tools for *in vitro* drug testing and basic cancer cell research.
